# Spiritual care in the intensive care unit. Is it already a reality?: an integrative review

**DOI:** 10.31744/einstein_journal/2025RW1081

**Published:** 2025-02-28

**Authors:** Alessandre Carvalho, Adriane Maria Netto de Oliveira, Camila Daiane Silva, Diéssica Roggia Piexak

**Affiliations:** 1 Programa de Pós-Graduação em Enfermagem Universidade Federal do Rio Grande Rio Grande do Sul RS Brazil Programa de Pós-Graduação em Enfermagem, Universidade Federal do Rio Grande, Rio Grande do Sul, RS, Brazil.

**Keywords:** Spirituality, Spiritual therapies, Critical care, Health personnel, Professional competence, Intensive care units

## Abstract

**Introduction:**

Spirituality has emerged as a phenomenon of interest in various global contexts. The adoption of spirituality as a fundamental aspect of healthcare remains underexplored, especially in critical environments such as intensive care units.

**Objective:**

To identify strategies for incorporating spiritual care into adult intensive care environments through an integrative literature review.

**Methods:**

An integrative review was conducted using the Embase, Web of Science, Medline/PubMed, PsycINFO, LILACS, and Cochrane Central databases. Twenty-one studies published in English, Spanish, or Portuguese over the last 10 years were selected.

**Results:**

Different approaches to spirituality were identified, including training implementation, individual interviews, meetings, educational programs, and practices involving healthcare professionals.

**Conclusion:**

Spirituality in the intensive care environment has been a growing reality in recent years through efforts aimed at helping healthcare professionals integrate spirituality into the care provided in clinical practice. Individual interviews were the primary strategy for incorporating spiritual care in intensive care unit settings. Questionnaires were used to support these interviews, and the frequency and duration typically involved a single session, ranging from 15 to 60 min.

## INTRODUCTION

Spirituality has proven to be a phenomenon of interest in various scenarios worldwide. One of the most widely accepted concepts of spirituality with international consensus is that of Puchalski. She defined spirituality as a dynamic and intrinsic aspect of humanity through which individuals seek meaning, purpose, and transcendence and experience their connection with themselves, others, family, community, society, nature, and the significant or sacred through their attitudes, habits, and practices.^1^

In the past decade, the topic of spirituality has been addressed in the intensive care unit (ICU) environment through the practices of professionals involved in patient care, thus characterizing spiritual care.^2,3^ This involves aspects related to promoting connections with others and investigating spiritual needs and religious beliefs. Although spiritual care has been implemented in some healthcare services, healthcare professionals often underestimate it, as its benefits are still relatively unexplored.^2^

One of the main barriers for professionals not considering and including spirituality in the care provided in ICUs is the lack of knowledge and time necessary for its practice, especially among doctors and nurses.^3-5^ Cultural context also appears to influence the incorporation of spirituality. Many European countries, especially more developed ones such as the United Kingdom, the Netherlands, and Germany, are more secularized than Asian or Middle Eastern countries. This may result in greater distance from incorporating the spiritual dimension into clinical practice, leading to less active and distant openness to integrating spirituality into healthcare.^6-9^

Despite the challenges related to implementing strategies that encompass spiritual care in ICUs, many patients and their families acknowledge that the feelings of vulnerability and stress arising from hospitalization require care that goes beyond the physical aspects. Accordingly, aspects of spirituality should also be considered.^2,4,9^

The ICU environment is characterized by acute changes in clinical aspects where many needs related to care become more evident, such as sleep deprivation, pain, fears, anxiety, distress, and feelings connected to life’s meaning, mortality, and hope, which are identified as spiritual needs.^10^

Considering the complexity and numerous specificities of the environment, spirituality is beginning to adapt to critical care settings by not only focusing on end-of-life care, from which it originally stems, but also as an approach integrated into the care provided throughout hospitalization.^11,12^

Regarding the needs of health care professionals, patients, and families in intensive care environments, the adoption of spirituality as a fundamental aspect of health care remains underexplored. This is particularly true of strategies that affirm their implementation within the care context. Therefore, this study aimed to identify strategies for incorporating spiritual care into the ICU through an integrative literature review.

## METHODS

The method employed was an integrative literature review selected to synthesize representative research addressing the theme of strategies for incorporating spiritual care into ICUs. The review followed the guidelines proposed by the Joanna Briggs Institute^13^ and was developed in accordance with the phases described by Toronto and Remington, who determined the following six stages of an integrative review: I) definition of the review question, II) literature search using predetermined criteria, III) critical evaluation of selected studies, IV) analysis and synthesis of literature, V) discussion of new knowledge, and VI) planning the dissemination of results.^14^ This approach was selected because of its systematic and rigorous nature, which facilitates the extraction of scientific studies with significant implications for clinical practice.^14^In addition, we adapted the study to follow the Preferred Reporting Items for Systematic Reviews and Meta-Analyses (PRISMA-ScR).^15^

### Search strategy

**Figure 1:**
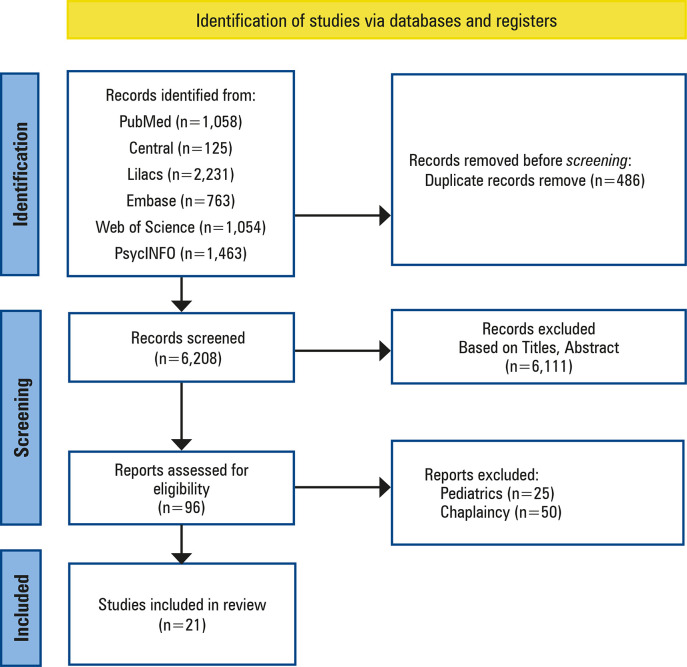
Path for the selection of articles included in the review

After formulating the research question, “What strategies have healthcare professionals in the ICU scientifically developed to include spirituality into patient care?” Articles were searched between April and November 2023 using the following online databases: Medline via PubMed, Cochrane Central, LILACS, PsycINFO, Web of Science, and Embase via Elsevier (Figure 1).

The search strategy utilized health descriptors (DeCS - *Descritores em Ciências da Saúde),* Medical Subject Headings (MeSH) terms, and keywords related to spirituality, healthcare professionals, and intensive care. These terms were combined using the Boolean operators AND and OR and adapted for each database (Table 1).

**Table 1:** Database search strategies

Databases	Search strategies
**PubMed**	#1 "Health Personnel"[MeSH] OR Personnel, Health OR Health Care Professional* OR HealthCare Provider* OR Healthcare Worker* #2 "Intensive Care Units"[MeSH] OR "Critical Illness"[MeSH] OR "Critical Care"[MeSH] OR intensive care OR close attention unit* OR respiratory care unit* OR intensive therap* OR critical care OR intensive treatment* OR critical* illn* OR sever* illn* OR serious* illn* OR ICU OR ITU #3"Spirituality"[MeSH] OR Spiritual* OR Chaplain* OR Pastoral Care OR Pastoral Counseling #4 #1 AND #2 AND #3
**Central**	#1 [mh "Intensive Care Units"] OR [mh "Critical Illness"] OR [mh "Critical Care"] OR intensive NEXT care OR close NEXT unit* OR respiratory NEXT care OR intensive NEXT therap* OR critical NEXT care OR intensive NEXT treatment* OR critical* NEXT ill* OR sever* NEXT ill* OR serious* NEXT ill* OR ICU OR ITU #2 [mh Spiritualism] OR Spiritual* OR Chaplain* OR Pastoral Care OR Pastoral Counseling #3 {AND #1-#2}
**LILACS**	#1 mh:”Pessoal de Saúde” OR mh:”Health Personnel” OR mh:”Personal de Salud” OR MH:M01.526.485$ OR MH:N02.360$ OR MH:SH1.030.020.020.010$ OR MH:VS3.004.001$ OR (TW:Personnel, Health) OR (TW:Health Care ProfessionaL*) OR (TW:HealthCare Provider*) OR (TW:Healthcare Worker*) #2 mh:”Unidades de Terapia Intensiva” OR mh:N02.278.388.493$ OR mh:”Cuidados Críticos” OR mh: E02.760.190$ OR mh:N02.421.585.190$ OR (TW:Cuidado Crítico) OR (TW:terapia intensiva) OR UTI OR UCI #3 mh:Espiritualidade OR mh:Spirituality OR mh:Espiritualidad OR Spiritual* OR Chaplain* OR Pastoral Care OR Pastoral Counseling OR MH:F02.880.705$ OR MH:K01.844.664.500$
**Embase**	#1 'intensive care unit'/exp OR 'intensive care':ab,ti OR 'close attention unit*':ab,ti OR 'intensive therap*':ab,ti OR 'critical care':ab,ti OR 'intensive treatment*':ab,ti OR 'critical* illn*':ab,ti OR ICU OR ITU #2 'religion'/exp OR Spiritual* OR Chaplain* OR 'Pastoral Care':ab,ti OR 'Pastoral Counseli':ab,ti #3 #1 AND #2#4 #3 AND [embase]/lim NOT ([embase]/lim AND [medline]/lim)Search strategy for Web of Science - Main collection by Clarivate Analytics #1 AB=("Intensive Care Units" OR "Critical Illness" OR "Critical Care" OR "intensive care" OR "close attention unit*" OR "respiratory care unit*" OR "intensive therap*" OR "critical care" OR "intensive treatment*" OR "critical* illn*" OR sever* illn* OR serious* illn* OR ICU OR ITU) #2 AB=("Spirituality" OR Spiritual* OR Chaplain* OR "Pastoral Care" OR "Pastoral Counseling")
**Web of Science**	#1 AB=("Intensive Care Units" OR "Critical Illness" OR "Critical Care" OR "intensive care" OR "close attention unit*" OR "respiratory care unit*" OR "intensive therap*" OR "critical care" OR "intensive treatment*" OR "critical* illn*" OR sever* illn* OR serious* illn* OR ICU OR ITU) #2 AB=("Spirituality" OR Spiritual* OR Chaplain* OR "Pastoral Care" OR "Pastoral Counseling")
**PsycINFO**	#1 Abstract: health care personnel OR Personnel, Health OR Health Care Professional* OR HealthCare Provider* OR Healthcare Worker* #2 Index terms: {Critical Illness} OR {Intensive Care} #3 Abstract: intensive care unit OR intensive care OR close attention unit* OR 'intensive therap* OR critical care OR intensive treatment* OR critical* illn* OR ICU OR ITU #4 Index terms: {Spirituality} OR {Spiritual Well Being} #5 Abstract: Spiritual* OR Chaplain* OR Pastoral Care OR Pastoral Counseling

### Inclusion criteria

The inclusion criteria were original studies and published articles available in full for complete text reading. When an article of interest was not freely accessible for full-text reading, the authors were contacted to access the material. Articles published in Portuguese, English, or Spanish in the past decade (between 2013 and 2023) were included. The exclusion criteria included review articles, theses, comments, abstracts published at events, and studies focusing on the pediatric/neonatal population and health chaplaincy.

## RESULTS

Twenty-one articles were selected (Table 2), originating from 10 countries: the United States of America, with 9 studies;^16-24^ Iran with three studies;^25-27^ Spain with two studies;^28,29^ and Singapore,^30^ Malaysia,^31^ Turkey,^32^ Colombia,^33^ Canada,^34^ Saudi Arabia,^35^ and the Czech Republic with one study each.^36^

**Table 2:** Characterization of the articles included in the review

Author (Year)	Country	Study design	Objectives	Study population	Evaluated outcomes
Canfield et al.^(^^16^^)^ 2016	USA	Qualitative content analysis study	Examine the care provided to the patient and their spiritual needs	Nursing team (n=30)	Definition of spirituality, spiritual needs
Choi et al.^(^^17^^)^ 2019	USA	Cross-sectional exploratory study	Determine how intensivist professionals address religious issues and the spiritual needs of patients and their families	Physicians (n=63), nurses (n=138), caregivers (n=18)	Religiosity and spiritual needs
Dunham et al.^(^^18^^)^ 2021	USA	Non-randomized clinical study	Investigate improvements related to a contemplation program (Spiritual Flow)	Nurses (n=228)	Well-being, stress, emotional states
Ernecoff et al.^(^^19^^)^ 2015	USA	Prospective multicenter cohort study	Determine the frequency with which healthcare professionals and family members discuss spirituality in clinical decision-making	Healthcare professionals (n=150), family members (n=546)	Content of religious and spiritual statements
Kincheloe et al.^(^^20^^)^ 2018	USA	Quasi-experimental descriptive study	Examine the effectiveness of a "spiritual care toolkit"	Nurses (n=54), family members (n=132)	Spiritual needs
Price et al.^(^^21^^)^ 2019	USA	Qualitative exploratory study	Identify challenges in providing end-of-life care for patients	Healthcare professionals (n=475)	Spiritual care, communication, decision-making, care satisfaction, ethics
Puchalski et al.^(^^22^^)^ 2020	USA	Multicenter exploratory descriptive study	Develop a curriculum for training healthcare professionals in spiritual care	Healthcare professionals (n=441)	Curriculum structured around domains involving spiritual care
Steinhauser et al.^(^^23^^)^ 2017	USA	Single-center randomized controlled trial	Determine the influence of an integrated care approach on critically ill patients	Patients (n=221)	Spiritual well-being, quality of life, anxiety, depression
Vesel et al.^(^^24^^)^ 2022	USA	Qualitative exploratory study	Explore healthcare professionals' perceptions of palliative care, leadership, and spiritual care during the COVID-19 pandemic	Healthcare professionals (n=25)	Recommendations, perceptions related to healthcare practice
Alimohammadi et al.^(^^25^^)^ 2018	Iran	Qualitative content analysis study	Explore the care needs of patients with severe TBI based on the perspective of nurses	Nurses (n=14)	Care-related needs
Riahi et al.^(^^26^^)^ 2018	Iran	Single-center randomized controlled trial	Investigate the effect of spirituality on the professional competence of nurses	Nurses (n=82)	Spiritual care competence
Salehi et al.^(^^27^^)^ 2020	Iran	Quantitative descriptive-analytical study	Determine attitudes toward spirituality and spiritual care among healthcare professionals	Healthcare professionals (n=298)	Spiritual care
García Torrejon et al.^(^^28^^)^ 2023	Spain	Multicenter descriptive observational study	Explore the perspectives of healthcare professionals, family members, and patients regarding the care provided for the relief of spiritual suffering	Healthcare professionals (n=655), Family members (n=340), Patients (n=216)	Spiritual needs, notions of spirituality, meaning, and purpose
de Diego-Cordero et al.^(^^29^^)^ 2022	Spain	Qualitative exploratory descriptive study with an ethnographic-phenomenological approach	Investigate the attitudes, knowledge, and perceptions of nurses	Nurses (n=19)	Competencies, perceptions, and knowledge about spiritual care
Yang et al.^(^^30^^)^ 2017	Singapore	Multicenter cluster-controlled study	Determine the effect of a spiritual care training program for healthcare professionals	Healthcare professionals (n=253)	Spiritual well-being, quality of life
Yik et al.^(^^31^^)^ 2021	Malaysia	Single-center randomized controlled trial	Investigate the effect of mindfulness practice on suffering and spiritual well-being in patients receiving palliative care	Patients (n=40)	Spiritual well-being, spiritual distress
Özakar et al.^(^^32^^)^ 2022	Türkiye	Cross-sectional descriptive study	Investigate the professional competence of nurses	Nurses (n=201)	Spiritual care competence
Hernández-Zambrano et al.^(^^33^^)^ 2020	Colombia	Qualitative study of the action-research type	Understand end-of-life care provided by healthcare professionals	Healthcare professionals (n=20)	Communication skills, decision-making, ethical issues, clinical management
Selby et al.^(^^34^^)^ 2017	Canada	Qualitative exploratory study	Identify opportunities to alleviate spiritual suffering through the opinions of healthcare professionals and patients	Healthcare professionals (n=21) Patients (n=16)	Definitions of topics related to spirituality and spiritual care
Albaqawi et al.^(^^35^^)^ 2017	Saudi Arabia	Quantitative correlational descriptive study	Examine the perception of holistic care	Nursing team (n=99)	Aspects of holistic care (physical, sociocultural, psychological, and spiritual)
Kisvetrová et al.^(^^36^^)^ 2016	Czech Republic	Cross-sectional descriptive study	Evaluate nurses' documentation regarding spiritual care and support	Nurses (n=277)	Frequency of biological, social, psychological, and spiritual activities

USA: United States of America; TBI: traumatic brain Injury; COVID-19: coronavirus disease 2019.

Regarding study design, the review identified seven qualitative studies,^16,21,24,25,29,33,34^ four randomized controlled trials,^23,26,30,31^ three cross-sectional studies,^17,32,36^ two quantitative studies,^27,35^ and one each of the following: non-randomized,^18^ cohort,^19^ observational,^28^ exploratory descriptive,^22^ and quasi-experimental studies.^20^ Regarding the population, 10 studies addressed multidisciplinary teams^17,19,21,22,24,27,28,30,33,34^ eight studies addressed only the nursing team,^16,18,25,26,29,32,35,36^ and three studies involved patients and family members in the context of intensive care.^20,23,31^

The primary methods for addressing and implementing spirituality in the ICU (Table 3) included individual interviews,^16,17,21,23-25,28,32,34^ trainings,^18,20,22,26,29-31^ “group awareness,”^33,35^ meetings and gatherings,^19^and record analyses.^36^ The predominant interfaces adopted were questionnaires,^17,19,21,27,28,32,34,35,36^ classes, practices, workshops,^22,23,24,26,29-31^ readings,^18,20^ question scripts,^16,25^ and textual construction.^28^

**Table 3:** Strategies for approaching/implementing spirituality in the intensive care unit

Author (Year)	Strategies	Interface	Frequency and duration	Key findings
Canfield et al.^(^^16^^)^ 2016	Individual interviews	Script of open-ended questions	A single moment	From a phenomenological perspective, the study provides a framework for creating resources to support nurses in intensive care and strategies to address the spiritual needs of patients
Choi et al.^(^^17^^)^ 2019	Intervention using validated S/R scales	Questionnaire involving attitudes/beliefs in addressing spiritual and religious needs	A single moment	Intensive care physicians recognize the importance of addressing the spiritual needs of patients, although a minority address such aspects in their clinical practice
Dunham et al.^(^^18^^)^ 2021	Reading and contemplation of books and meditation practices	Book and meditation exercises	1 training - Follow-up for 2 months	The 'Spiritual Flow' program was associated with an improvement in the well-being of nurses
Ernecoff et al.^(^^19^^)^ 2015	Meeting with family members and healthcare professionals	Paper questionnaire and audio recording	Follow-up for 3 years in 13 ICUs	Healthcare professionals rarely explore S/R issues, observed in less than 20% of cases
Kincheloe et al.^(^^20^^)^ 2018	Implementation of a “spiritual care toolkit”	Books, newspapers, CDs, DVDs, crosses, rosaries	1 training - Follow-up for 13 weeks	The toolkit has the potential to help meet the spiritual needs of patients and families. However, successful implementation requires support and funding from the institution
Price et al.^(^^21^^)^ 2019	Online individual interviews	Questionnaire with open-ended questions	1 time assessment - Data collection over 12 weeks	The concerns were divided into seven themes: communication (97%), decision-making (75%), education needs (60%), end-of-life care (48%), ethics (24%), satisfaction with care (9%), spiritual sensitivity (6%)
Puchalski et al.^(^^22^^)^ 2020	Development of an interdisciplinary curriculum for spiritual care	In-person classroom and online training	3 days of training (23 h) - Follow-up for 1 year	The curriculum proved to be suitable for different clinical settings, being offered in an interprofessional manner
Steinhauser et al.^(^^23^^)^ 2017	Structured interview focusing on forgiveness, life review, legacy, and heritage issues	Online interviews and relaxation sessions using music	3 sessions of 45 min each over 1 month	The intervention group had a greater impact on social well-being. However, it did not show improvement in anxiety, depression, and quality of life indices compared with the control group
Vesel et al.^(^^24^^)^ 2022	Semi-structured interview	Videoconference	1 session of 35 min	The respondents recognized that the role of palliative care increased during the pandemic, contributing to the efficiency of hospital services
Alimohammadi et al.^(^^25^^)^ 2018	Individual interviews	Semi-structured interview (in-person or by phone)	A single moment (30-120 min)	Patients with TBI have various care needs in physical, psychosocial, and spiritual dimensions. Healthcare teams should be attentive to address these care needs
Riahi et al.^(^^26^^)^ 2018	Spiritual intelligence protocol	Workshop, validated questionnaires, and meditation practices	8 sessions of 90 min each over 8 weeks	Spiritual intelligence training positively affected the spiritual care of nurses, where 89% of them had not received any previous training in the subject of spirituality
Salehi et al.^(^^27^^)^ 2020	Validated questionnaire	Online (e-mail, communication app)	A single moment	Attitudes toward spirituality were directly and significantly related to spiritual care
García Torrejon et al.^(^^28^^)^ 2023	Online individual interviews	Online semi-structured questionnaires	1 assessment - Follow-up for 9 months in 41 ICUs	The majority (69.7%) of professionals consider spirituality as part of care, but half (50.1%) of them did not feel competent in providing spiritual care, and the majority (83.4%) considered training in this area necessary
de Diego-Cordero et al.^(^^29^^)^ 2022	Quantitative instruments and expert panel experience	Interview script supported by an expert panel	A single moment (50-60 min)	Healthcare professionals working in the ICU should consider spirituality in moments of crisis. However, the lack of training, time, and workload are barriers to providing spiritual care
Yang et al.^(^^30^^)^ 2017	Spiritual care training program and validated questionnaire	Class with group discussion	One - 30-min training session	A brief spiritual care training program may help with quality of life, but no significant effect on the participants' well-being was observed
Yik et al.^(^^31^^)^ 2021	Mindfulness practice and validated questionnaire	Semi-structured questionnaires and audio recording	One - 5-min session	Five minutes of mindfulness practice significantly reduced the sense of suffering compared with the control group. A brief 5-min mindfulness exercise proved effective in promoting immediate relief of suffering and improved spiritual well-being
Özakar et al.^(^^32^^)^ 2022	Individual interviews	Validated questionnaire	A single moment (15-20 min)	Nurses with high scores on the spiritual competence scale were able to diagnose and address the spiritual needs of their patients
Hernández-Zambrano et al.^(^^33^^)^ 2020	Awareness of end-of-life care and its approaches	Construction of texts in the form of narratives (1,800-2,500 words)	4 sessions of 2 h each in each ICU	Healthcare professionals consider preserving the quality of life as a therapeutic goal during the ICU stay through personalized care to respect the diverse needs of the patient
Selby et al.^(^^34^^)^ 2017	Individual interviews	Semi-structured questionnaires and audio recording	A single moment	Many discrepancies in the perception of definitions of spirituality, spiritual care, and spiritual distress were observed, which may lead to healthcare professionals facing difficulty in providing spiritual care
Albaqawi et al.^(^^35^^)^ 2017	Awareness of five topics comprising holistic care	Structured questionnaire	A single moment	Most nurses were aware of the aspects comprising holistic care and suggested an orientation and enhancement program for nurses to improve their practice
Kisvetrová et al.^(^^36^^)^ 2016	Analysis of nursing records	Structured questionnaire	Data collection over 14 months	The psychosocial and spiritual dimensions had the lowest nursing records. Support and education on actions in these dimensions can increase nurses' competence in communication related to end-of-life processes with family members and patients

TBI: traumatic brain injury; S/R: spirituality/religiosity; ICU: intensive care unit.

Concerning the frequency and duration of these strategies, most studies occurred only at a single moment,^16-21,24,25,27-32,34,35^ two studies had three sessions,^22,23^ one study had four sessions,^33^ one study had eight sessions,^26)^ and one study did not have a regular frequency as it involved record analysis.^36^

## DISCUSSION

Within the field of health sciences, the relationship between spirituality and care has begun to emerge. This relationship has been particularly highlighted in the context of severe illnesses, especially in patients with cancer, marking a significant milestone in the evolution of palliative care.^37^ This important approach has led to more in-depth studies of spirituality in recent decades in the context of this type of care and within this specific population.

Since the beginning of the 21st century, with the growing interest in researching spirituality in various settings, attention to care has expanded to several areas, including intensive care.^2,3,9,16^ Recently, the approach to spiritual care in ICU settings has become a perceived reality in various countries, with the involvement of several healthcare professionals in the care practice.^17,28,30,31,36^

The role of nursing as a science dedicated to investigating healthcare, its interface with the spiritual dimension in the ICU, and its role and actions in spiritual care are worth highlighting. Many studies have specifically addressed the role of nurses as primary interlocutors of care.^16,18,25,26,29,32,35,36^ Nurses are fundamental professionals in critical care settings, whereas other multidisciplinary team members form a clinical body that shares competencies attributed to caregiving.

Although care is provided by nurses from the perspective of comprehensiveness, all professionals are responsible for including different dimensions of care in their care practices.^38^ The integration of spirituality into the clinical practice of intensive care still poses some challenges, ranging from a lack of knowledge and time for its application to the discomfort experienced by professionals.^6,22,32,34^

To initiate a culture where spirituality is integral to the care plan within the intensive care environment, implementing pedagogical and formative actions, as well as strategies that bring this knowledge closer to intensive care health professionals, is necessary. Some educational initiatives in critical care units indicate that institutions are increasingly incorporating spiritual care into their practices.^18,20,26,33^

Individual interviews have been the most common strategy for bringing the concept of spirituality closer to intensive care professionals, aiming to standardize their knowledge on this topic.^16,21,23-25,28,32,34^ Interviews have proven effective in raising awareness among healthcare professionals about various topics related to the spirituality/religiosity (S/R) dimension, such as the definitions and concepts of spirituality, spiritual needs and care, quality of life, and professional competencies. This strategy is particularly useful for acquiring knowledge that is not yet widely disseminated in intensive care settings.

Individual interviews, training sessions, meetings, and gatherings share a generic format that allows professionals to be educated and sensitized to existential issues. These issues often arise due to the extreme vulnerability experienced by patients facing the various challenges inherent in hospitalization in critical care units.^2,4,9^ However, for professionals to recognize, assess, and address spiritual needs, these educational strategies must be well-structured to encompass the various spirituality-related constructs within the clinical context and align with other healthcare approaches that constitute good clinical practices.

Among the educational interventions on spirituality, it is evident that while various strategies have expanded the themes around spiritual care, there is also a noticeable lack of protocols for addressing this subject. This inconsistency in managing the spiritual dimension in intensive care can be attributed to the diverse approaches to spirituality within clinical settings and the lack of a methodological structure to encompass the S/R dimension.

Some strategies incorporate spiritual practices such as meditation, the use of sacred texts, symbols, and music, and mindfulness-associated breathing exercises. Creating a common curriculum for training different healthcare professionals that addresses, assesses, and recognizes the spiritual needs of patients is a great example of strengthening and decentralizing care for interprofessional teams.^20,22,23,26,31^

The multiple interfaces for transmitting this knowledge, whether in-person^17,18,20,29,33^ or remotely,^21-24,28^ show how much this topic has adapted to modern methodologies to facilitate its dissemination. However, no consensus has been reached regarding the most suitable interface for introducing and implementing spiritual care, highlighting the need to explore the most effective methodologies for addressing spirituality in intensive care settings.

Numerous studies have addressed spirituality at a single point in time, focusing on various implementation strategies or training.^16-21,24,25,27-32,34,35^ This also highlights the need for a regulatory body of experts to establish defined hours for spiritual care.

Spirituality, as a dimension of care in intensive care settings, is a complex and relatively recent topic that requires significant development to become effectively integrated into care guidelines and protocols. International organizations and private agencies that accredit quality related to good practices recognize the importance of spirituality in the hospital environment and how it forms part of the care plan.^39-41^ However, institutional interest is still required for its implementation, operationalization, and effectiveness.^20^

### Limitations

Although robust studies with good methodological designs in the area of spirituality and health are available, the intensive care environment still has a limited number of randomized controlled trials, restricting the generalizability of the conclusions.

## CONCLUSION

Spirituality in the intensive care environment has intensified in recent years, thanks to initiatives that assist healthcare professionals integrate spirituality into clinical practice**.** The primary strategy for incorporating spiritual care in intensive care unit settings was individual interviews to familiarize intensive care professionals with the concept of spirituality. These interviews can effectively address aspects related to spiritual care and facilitate its incorporation in intensive care unit settings.

Introducing the theme of spirituality to the intensive care environment, where existential situations oscillate between intense suffering, life, and death, as well as various difficulties arising from hospitalization, highlights the relevance of including spirituality in care to alleviate negative feelings, specifically in intensive care units. It also highlights the responsibility of professionals to adopt practices encompassing the entirety of care provided, which is a fundamental aspect of good clinical practice.
